# Neuroprotective effects of ammonium tetrathiomolybdate, a slow-release sulfide donor, in a rodent model of regional stroke

**DOI:** 10.1186/s40635-020-00300-8

**Published:** 2020-04-09

**Authors:** Bruna Pescador Mendonça, Juliano Dos Santos Cardoso, Monique Michels, Ana Carolina Vieira, Diogo Wendhausen, Andressa Manfredini, Mervyn Singer, Felipe Dal-Pizzol, Alex Dyson

**Affiliations:** 1 Laboratory of Experimental Pathophysiology, University of Southern Santa Catarina, Criciúma, Brazil; 2 Bloomsbury Institute for Intensive Care Medicine, Division of Medicine, University College London, Gower St, London, WC1E 6BT UK

**Keywords:** Ischemia, Reperfusion, Brain, Oxidative stress, Reactive oxygen species, Mitochondria, Cytochrome C oxidase

## Abstract

**Background:**

Several therapeutic strategies to rescue the brain from ischemic injury have improved outcomes after stroke; however, there is no treatment as yet for reperfusion injury, the secondary damage caused by necessary revascularization. Recently we characterized ammonium tetrathiomolybdate (ATTM), a drug used as a copper chelator over many decades in humans, as a new class of sulfide donor that shows efficacy in preclinical injury models. We hypothesized that ATTM could confer neuroprotection in a relevant rodent model of regional stroke.

**Methods and results:**

Brain ischemia was induced by transient (90-min) middle cerebral artery occlusion (tMCAO) in anesthetized Wistar rats. To mimic a clinical scenario, ATTM (or saline) was administered intravenously just prior to reperfusion. At 24 h or 7 days post-reperfusion, rats were assessed using functional (rotarod test, spontaneous locomotor activity), histological (infarct size), and molecular (anti-oxidant enzyme capacity, oxidative damage, and inflammation) outcome measurements. ATTM-treated animals showed improved functional activity at both 24 h and 7-days post-reperfusion, in parallel with a significant reduction in infarct size. These effects were additionally associated with increased brain antioxidant enzyme capacity, decreased oxidative damage, and a late (7-day) effect on pro-inflammatory cytokine levels and nitric oxide products.

**Conclusion:**

ATTM confers significant neuroprotection that, along with its known safety profile in humans, provides encouragement for its development as a novel adjunct therapy for revascularization following stroke.

## Introduction

Stroke is the third leading cause of death in developed countries [1], the principal cause of severe long-term disability worldwide [2], and the largest contributor to global neurological disability-adjusted life-years [3]. Advances such as thrombolytic therapy and angioplasty have revolutionized acute management [4, 5]; however, this obligatory revascularization induces further injury through reperfusion. This reperfusion injury is caused by various putative mechanisms including oxidative stress and damage, metabolic derangements, and inflammation [6–10]. Additional therapeutics geared towards attenuation of reperfusion injury could provide further impact on both short- and long-term outcomes [11–13].

Hydrogen sulfide (comprising gaseous H_2_S and anionic HS^−^; herein collectively referred to as sulfide) is the third endogenous “gasotransmitter” alongside nitric oxide and carbon monoxide [14]. It is produced endogenously either enzymatically (from l-cysteine) or by reduction of its oxidized forms (e.g., sulfite/sulfate), and plays an important role as a signaling molecule across numerous physiological systems [15]. Our interest in sulfide as a therapeutic relates to its ability to transiently inhibit mitochondrial cytochrome C oxidase [16]. This, and subsequent reduction of mitochondria-derived reactive oxygen species production, improves outcomes in preclinical injury models [17–19]. However, despite over 10 years of promising preclinical research, no sulfide-based therapies have yet proven successful in randomized Phase 2/3 clinical trials [20]. Initially, simple sulfur salts, e.g., sodium sulfide (Na_2_S) were used; however, these salts “generate” sulfide in a near-instantaneous (and hence, uncontrolled) fashion; this raises safety concerns that likely precluded their clinical development. Subsequently, drug design of sulfide mimetics gained sophistication, with characterization of several classes of sulfur-hybrid molecules and slow-release sulfide “donors”. These enable more controlled sulfide release (to more accurately reflect those derived from endogenous sources) and better targeting to the intended (intracellular) site of action [21].

Ammonium tetrathiomolybdate (ATTM; [NH_4_]_2_MoS_4_) is a copper chelator historically used for the treatment of Wilson’s disease and, more recently, has shown some efficacy for cancer [22]. Constituting a transition metal (molybdenum) core and four covalently bound sulfur atoms, scission of the metal-sulfur bonds enables this drug to act as a slow-release sulfide donor [23, 24]. We have recently characterized its chemistry and mode of cellular uptake [25], and demonstrated both decreased global aerobic respiration and protective effects in several models of ischemia/reperfusion injury [23]. Our working hypothesis is that rapid substrate provision necessarily provided by revascularization drives mitochondrial respiration to a level that generates large quantities of damaging ROS [26]. By modulating this response with a short-term inhibitor of mitochondrial cytochrome C oxidase, we contend that we can achieve a level of metabolism that supports cell viability and functionality, along with some amelioration of overproduction of ROS. We have provided mechanistic evidence elsewhere to support these claims [23]. In this study, we wished to further explore the therapeutic potential of ATTM, and to better understand the downstream effects of its action in revascularized tissue. We utilized a rodent focal brain injury model and hypothesized that ATTM, given at reperfusion, would improve both functional and histological outcomes after stroke.

## Materials and methods

### Drugs and reagents

ATTM was purchased from Sigma-Aldrich (Saint Louis, MO, USA) and prepared as previously described [23]. A certificate of analysis can be found here: [27]. Material was sought from this specific supplier as we have previously observed good consistency between batches [23]. We subject each batch of ATTM to an in vitro sulfide-release test as this is the principal pharmacological mechanism we seek to exploit. A full description of this methodology is available elsewhere [25]. If using ATTM for this purpose, we would encourage other researchers to establish this assay and provide quality control data on the particular batch utilized. Material used in this study was within our predefined criteria; we could detect between 3 and 4 ppm of H_2_S following standard incubation conditions (1 h at physiological pH and temperature). Unless otherwise stated, all other materials were purchased from Sigma-Aldrich.

### Animals and husbandry

Experimental procedures involving animals were in accordance with the Brazilian legislation (CONCEA; Council for the Control of Animal Experimentation), and performed following approval from the University of Southern Santa Catarina ethics committee. The manuscript adheres to ARRIVE (Animal Research: Reporting of In vivo Experiments) guidelines. Two-month old male Wistar rats (approximately 300 g body weight) were used in all experiments, bred in-house at the University of Southern Santa Catarina (Criciúma, Brazil). All animals were in good health, certified pathogen-free, and housed in cages of four individuals on a 12-h light/dark cycle. Food and water were provided ad libitum. Standard cages and bedding were used. Tissue paper and cardboard tubes provided additional comfort and cage enrichment. Experiments were started at 8 AM local time. We used rats as we have found they more closely mimic human metabolic responses; mice rapidly reduce their metabolism in response to systemic challenges [28]. We thus considered that use of mice would hinder a translational evaluation of a molecule that modulates metabolism. At experiment end, all animals were euthanized by terminal anesthesia (intraperitoneal sodium pentobarbitone, Cristália, São Paulo, Brazil).

**Fig. 1:**
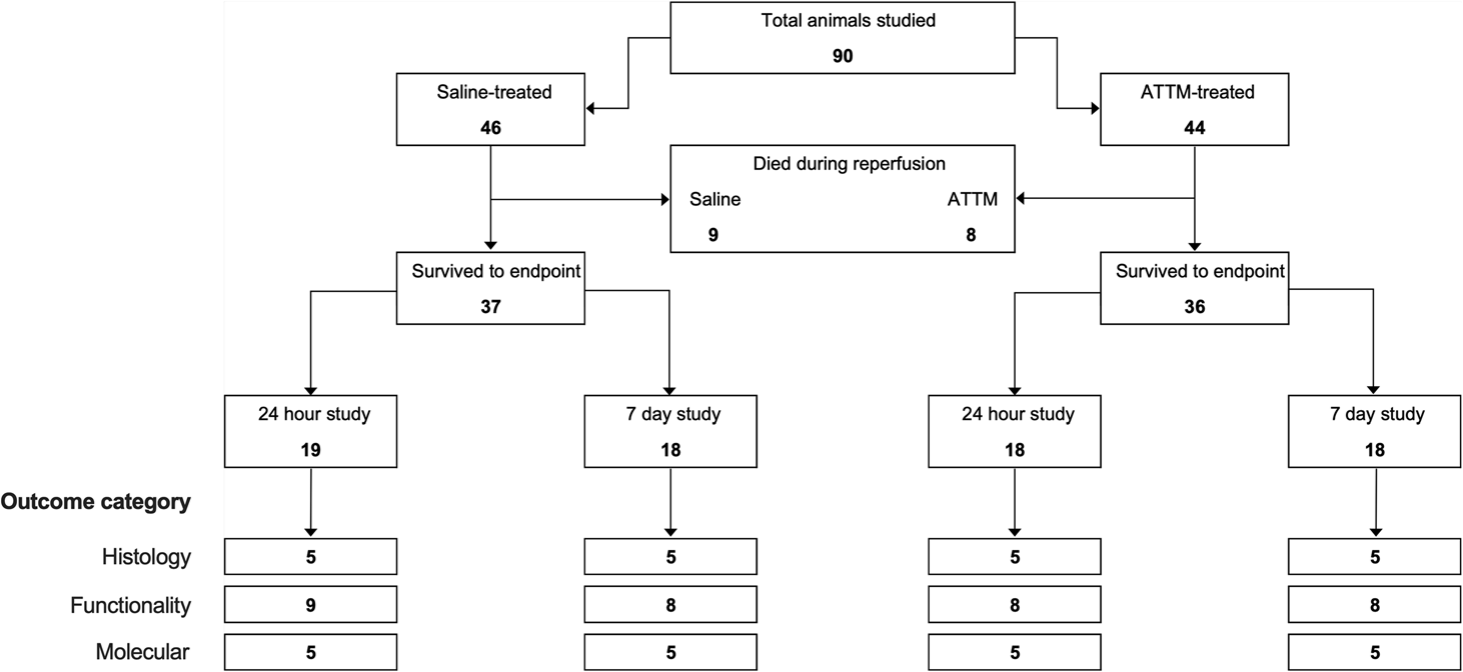
Experimental design. Flow diagram depicting the number of animals used for each category of outcome measure

We used separate animals for the following outcome measures described in detail below: (i) infarct size, (ii) behavioral tests, and (iii) molecular outcome measures (Fig. 1). Use of separate animals for these assessments prevented any potential modification of our outcomes by a prior test or training procedure. We used 90 animals in total that were divided into two time points studied, and the three categories of outcome measure. We aimed for a minimum of 5 animals per group for histological and molecular outcome measures and 8 per group for behavioral tests. Using fixed numbers of this magnitude has previously enabled us to demonstrate clinically and statistically relevant differences [23, 29].

### Stroke induction

Animals were subjected to transient middle cerebral artery occlusion (tMCAO), as shown and described in detail elsewhere [30]. Briefly, animals were anesthetized with isoflurane in room air: 5% for induction, 2% for surgery, and 1–2% for maintenance. Asepsis (chlorhexidine, 4%) applied to the neck was followed by a 2 cm incision in the ventral cervical region. Local anesthetic (0.5% xylocaine) was injected subcutaneously throughout the incision site. The right internal jugular vein was cannulated using 0.96-mm external diameter PVC tubing (Scientific Commodities Inc., Lake Havasu City, AZ, USA). The left common carotid artery (CCA), the internal carotid artery (ICA), the occipital artery (OA), and the external carotid artery (ECA) were isolated. The middle cerebral artery (MCA) was occluded for 90 min by advancing (until perceived resistance) a 4-0 nylon suture monofilament with a rounded silicone tip (Doccol Corporation, MA, USA) through the ICA. Core temperature was maintained at 37 °C using a rectal probe and heated mat (Harvard Apparatus, Cambridge, Cambs, UK).

### ATTM treatment

Immediately prior to reperfusion, animals were randomly assigned to receive an intravenous bolus of ATTM (10 mg/kg), or an equivalent volume of saline (2 ml/kg), administered over 1 min. The intraluminal wire was then removed from the MCA. Animals received a further intravenous infusion of ATTM (10 mg/kg/h) or an equivalent volume of saline (10 ml/kg/h) over 60 min. Anesthesia was maintained throughout ischemia and up until 1-hour post-reperfusion. The jugular venous line was then permanently occluded and the surgical site closed. Analgesia was administered immediately and at 12 h post-operatively (dipyrone, 80 mg/kg subcutaneously, Sanofi-Aventis, Suzano, São Paulo, Brazil). Animals were allowed to recover until further assessment at either 24 h or 7 days post-reperfusion. At the time of treatment, blinding could not occur due to the distinct coloration of ATTM and the obvious smell of sulfide from vials containing dissolved material. However, all subsequent behavioral, histological, and biochemical assessments were performed by investigators unaware of the treatment allocation. We selected the quantity of ATTM administered here as this has previously conferred protection following myocardial and (global) cerebral ischemia, and hemorrhage/resuscitation [23]. Additionally, ATTM does not impact on global hemodynamics or cardiac function (mean arterial blood pressure, cardiac output, and contractility) at this dose level [23].

### Lesion area

To measure infarct size, rats were terminally anesthetized and perfused with ice-cold phosphate-buffered saline. Brains were removed and sliced into 2-mm coronal sections, and the area of the ischemic lesion (as a percentage of total brain area) was measured using 2,3,5-triphenyltetrazolium chloride (TTC; 0.1% w/v)-stained sections and the ImageJ analysis software (National Institutes of Health, Bethesda, MD, USA). The TTC method has been evaluated for use in this setting [31].

### Behavioral tests

At 24 h or 7 days post-reperfusion, animals were assessed using functional behavioral assays. Motor coordination and balance were evaluated by the rotarod test. The apparatus consists of a rotating bar (8 rpm) on which the animal was positioned. Latency to the first fall was recorded with a cut-off time of 5 min. Locomotor and exploratory activity were assessed using an open field test. The apparatus comprised a 40 × 60-cm arena surrounded by 50-cm high walls, with the floor divided into nine rectangles. Animals were placed in the left rear quadrant and allowed to explore the arena for 5 min. Line crossings and rearing were counted, and used as an index of spontaneous locomotor activity.

### Molecular outcomes

At 24 h or 7 days post-reperfusion, animals were euthanized, the ipsilateral brain cortex was isolated, and antioxidant enzyme capacity, oxidative damage, proinflammatory markers, and nitric oxide products were measured. All assays were performed in duplicate. Formation of thiobarbituric acid-reactive substances (TBARS) during an acid-heating reaction was used as an index of oxidative damage to lipids [32]. Briefly, samples were mixed with 1 mL 10% trichloroacetic acid and 1 mL 0.67% thiobarbituric acid and then boiled in a water bath for 15 min. Malondialdehyde (MDA) equivalents were determined by measuring absorbance at 535 nm using 1,1,3,3-tetramethoxypropane as an external standard. Results are expressed as MDA equivalents per milligrams of protein. Protein concentration was determined by the Lowry method [33]. Oxidative damage to proteins was assessed by determination of carbonyl groups based on the reaction with dinitrophenylhydrazine [34]. Briefly, proteins were precipitated by the addition of 20% trichloroacetic acid, re-dissolved in 10 mmol/l dinitrophenylhydrazine, and absorbance read at 370 nm. Results were expressed as protein carbonyls per milligrams of protein. Catalase activity was determined on the disappearance of hydrogen peroxide at 240 nm in a reaction medium containing 20 mmol/L H_2_O_2_, 0.1% Triton X-100, 10 mmol/L potassium phosphate buffer, and 0.1–0.3 mg protein/mL at pH 7.0 [35]. One CAT unit was defined as 1 mol of hydrogen peroxide consumed per minute. Activity was reported as CAT units per milligrams of protein. Superoxide dismutase (SOD) activity was measured by the inhibition of autoxidation of adrenaline. A calibration curve was performed using purified SOD as a standard to calculate the specific activity of SOD present in the test samples. This activity is represented as units of SOD activity per milligrams of protein [36]. Cytokine assays (TNF-α, IL-1β, IL-6), determined by ELISA (DuoSet, R&D Systems, MN, USA) were performed using kits according to the manufacturers’ instructions. Finally, nitrite/nitrate concentration was assayed spectrophotometrically using a Griess reagent (1% sulfanilamide in 5% phosphoric acid and 0.1% N-1-naphthylethylenediamine dihydrochloride in twice distilled H_2_O) and vanadium (III) chloride [37]; optical density at 550 nm was measured.

### Statistical analyses

Data are presented as median, interquartile range, and range. As different animals were used for the 24 h and 7-day time points, all statistical analyses of treatment effects were performed using non-parametric unpaired (Mann-Whitney) *T* tests. All analyses were two-tailed and performed using GraphPad Prism (version 7.0d, GraphPad Software, San Diego, CA, USA). *p* values < 0.05 were considered statistically significant.

## Results

**Fig. 2:**
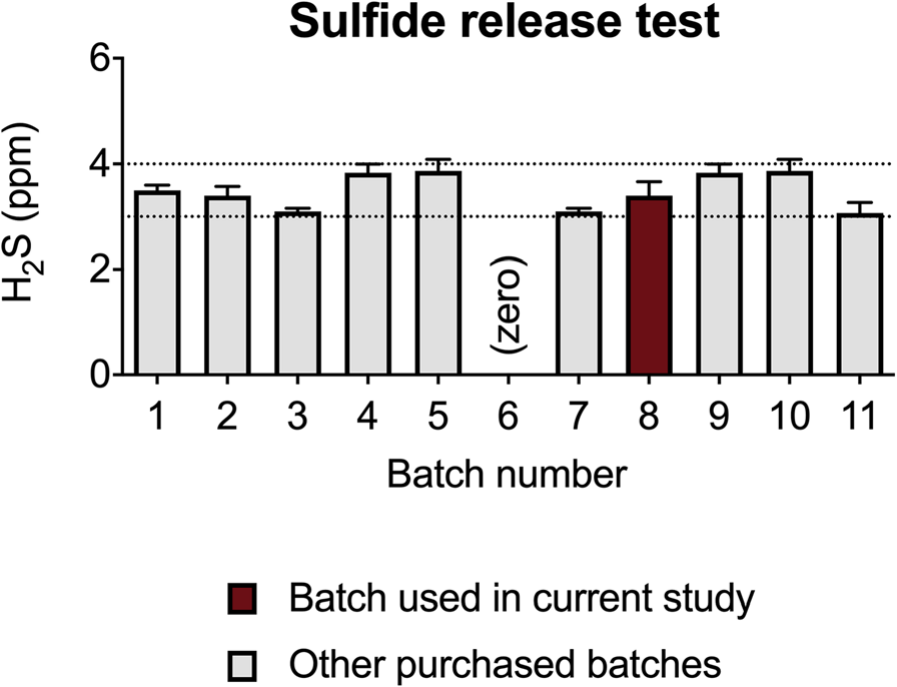
Sulfide release*.* Comparison of *Batch 8* used in the current study against previous and subsequent batches purchased from Sigma-Aldrich. *Batch 8* was deemed suitable for use having aligned with our predefined criteria (3–4 parts per million; ppm, denoted by the dotted lines). Note that *Batch 6* was purchased elsewhere and does not release its sulfur as sulfide; this underpins the importance of establishing this assay, if used for this purpose

The batch of ATTM we used in this study conformed to our predefined criteria for sulfide release (Fig. 2).

Of the 90 animals studied, 17 died during early reperfusion (i.e., within the first hour while still under anesthesia). Ten animals died suddenly following overwhelming focal ischemia and revascularization, and 7 animals from anesthesia-induced airway obstruction. No deaths were deemed to be drug-related as a similar number of animals from each group succumbed to early demise (9 saline-treated vs 8 ATTM-treated; Fig 1).

**Fig. 3:**
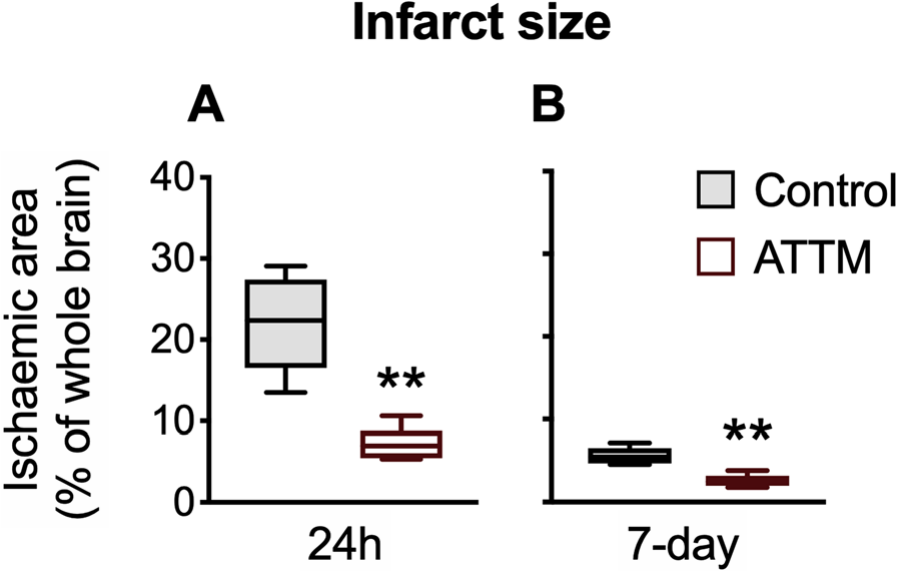
Histological outcomes. Brain infarct size, determined at 24 h or 7 days post-reperfusion, is shown in panels **a** and **b**, respectively. *n* = 5 per group. **p < 0.01 vs controls, unpaired *T* test

Administration of ATTM at reperfusion provided significant neuroprotection with relative reductions in infarct size of 68% at 24 h (*p* < 0.01) and 54% at 7 days (*p* < 0.01) post-reperfusion (Fig. 3). Notably, there was an improvement in both groups between 24 h and 7 days post-insult. A representative selection of original brain slices is shown in Supplementary Fig 1.

**Fig. 4:**
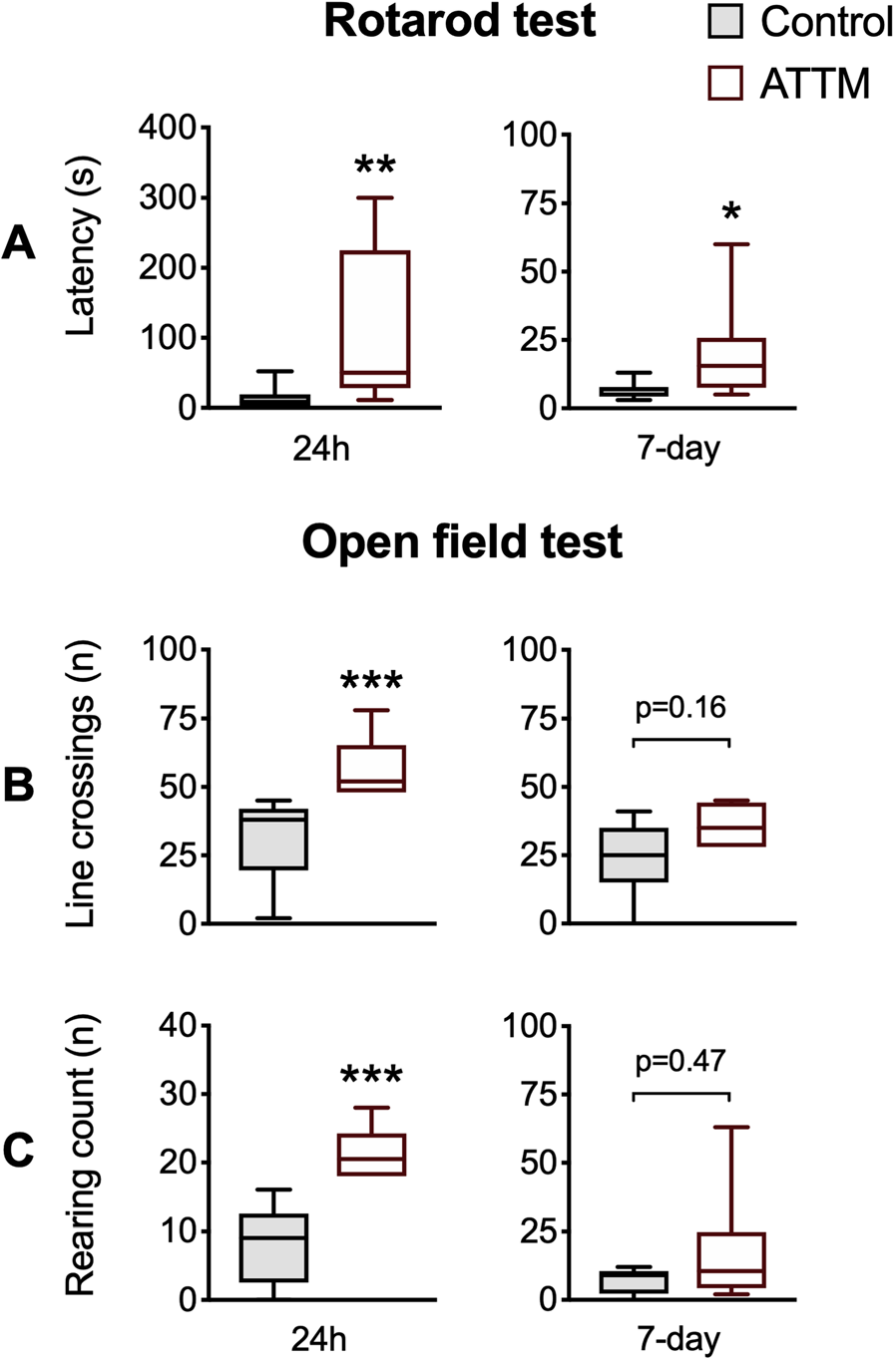
Motor performance and locomotor activity. Motor performance assessed by rotarod is shown in panel **a**. Spontaneous line crossings and rearing counts using the open field test are shown in panels **b** and **c**, respectively. *n* = 8–9 per group. *p < 0.05, **p < 0.01, ***p < 0.001 vs controls, unpaired *T* test

We next investigated whether this histological improvement with ATTM treatment translated to superior functional outcomes, assessed using motor coordination and spontaneous locomotor activity tests. The latency to first fall in the rotarod task was significantly (*p* < 0.05) improved by ATTM treatment at both 24 h (*p* < 0.01) and 7 days (*p* < 0.05) post-reperfusion (Fig. 4a). At 24 h, an assessment of spontaneous locomotor activity revealed that ATTM-treated animals performed twice the number of line crossings and rearing events compared to their placebo-treated counterparts (Fig. 4b, c). By 7 days post-insult, these differences did not differ between treatments (Fig. 4b, c).

**Fig. 5:**
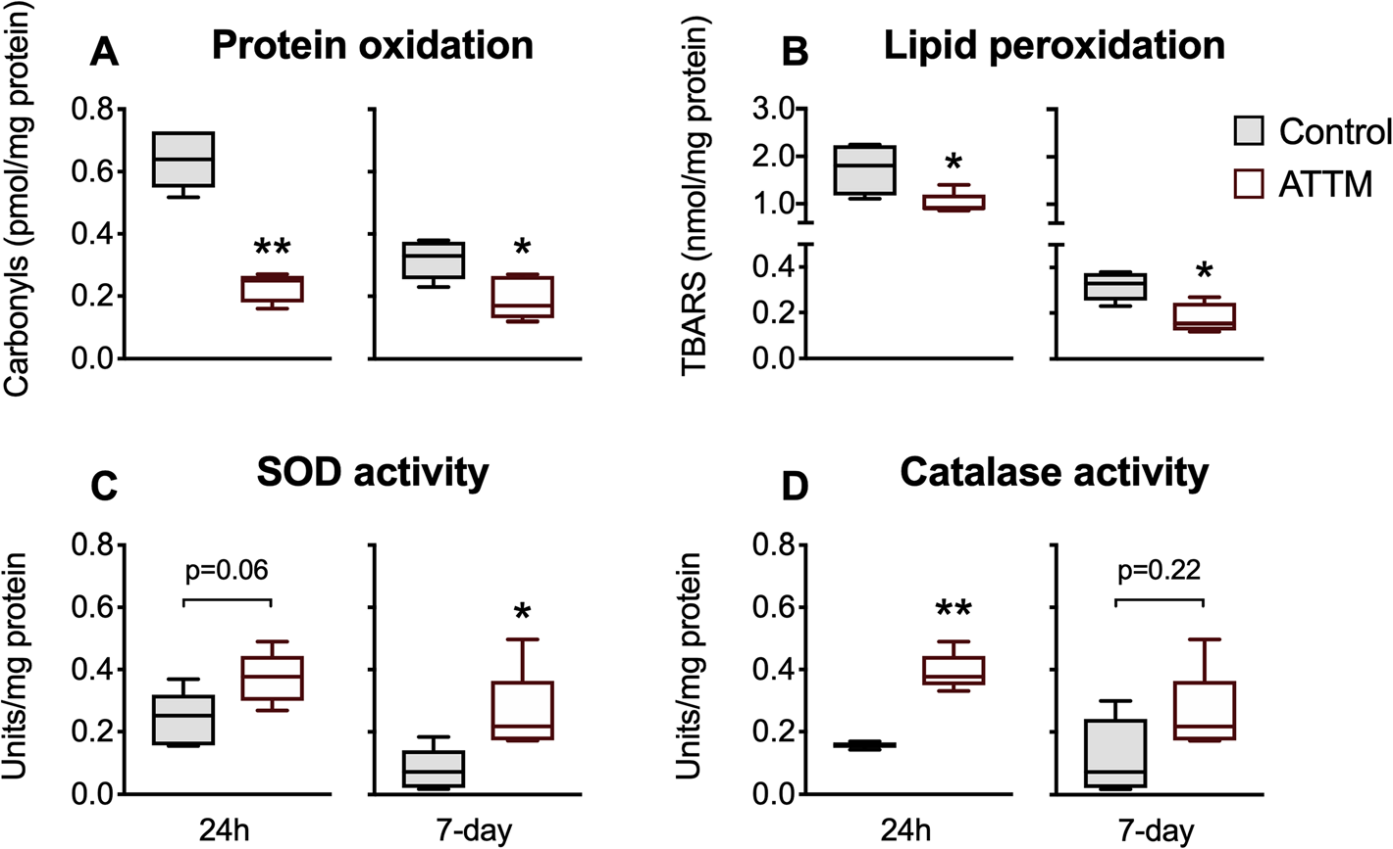
Brain oxidative damage and anti-oxidant enzyme capacities. Oxidative damage to proteins and lipids measured by carbonyl and TBARS formation are shown in panels **a** and **b**, respectively. Enzyme activities of superoxide dismutase and catalase are shown in panels **c** and **d**, respectively. *n* = 5 per group. *p < 0.05, **p < 0.01 vs controls, unpaired *T* test. SOD, superoxide dismutase; TBARS, thiobarbituric acid reactive species

**Fig. 6:**
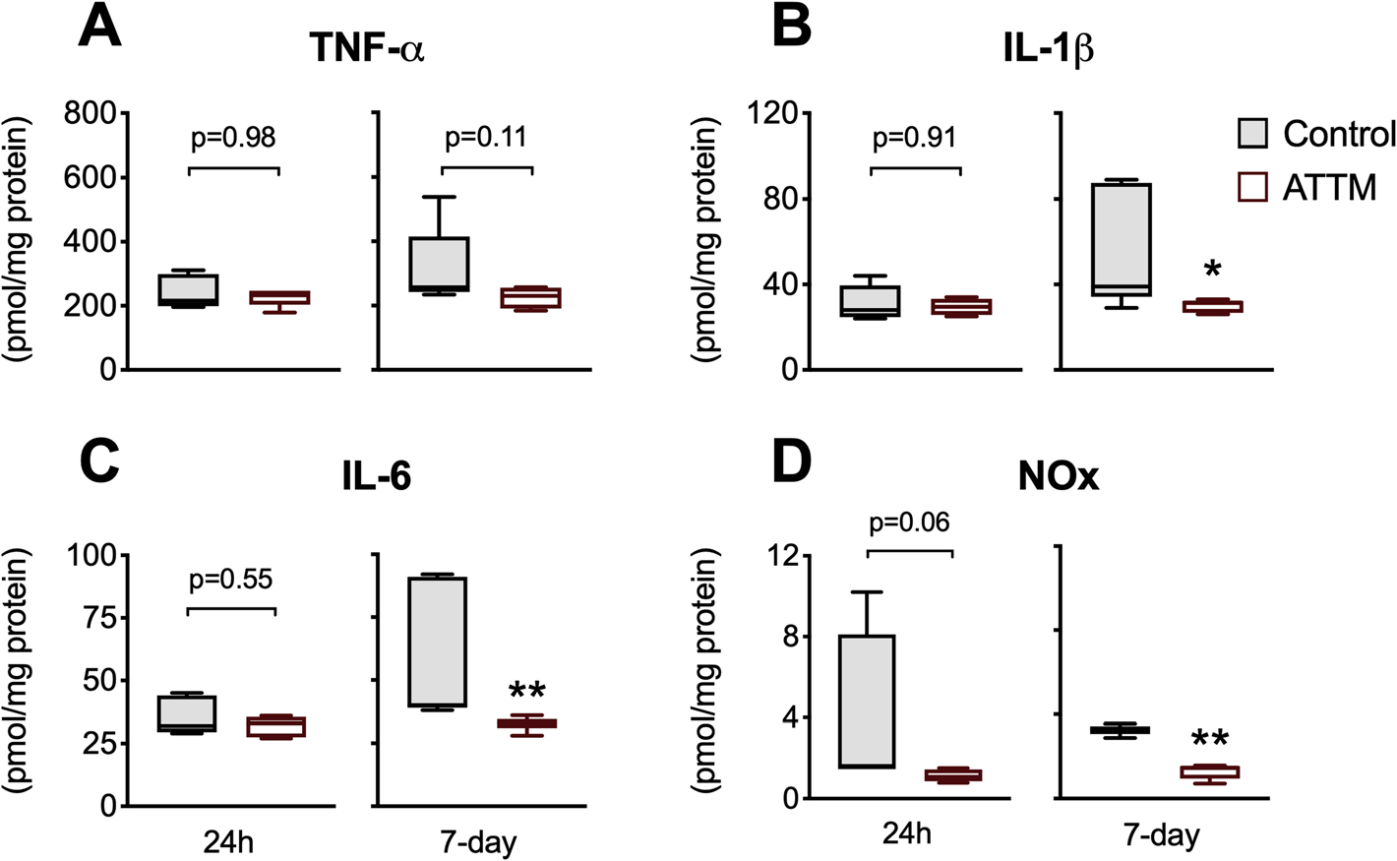
Pro-inflammatory markers. Panels **a***–***d** depict TNF-α, IL-1β, IL-6, and total nitric oxide (NOx) products, respectively. *n* = 5 per group. *p < 0.05, **p < 0.01 vs controls, unpaired *T* test

We studied downstream molecular changes in brain tissue that could point to putative mechanisms that explain the neuroprotective effects of ATTM. Attenuation of oxidative damage to proteins (carbonyls; Fig. 5a) and lipids (TBARS; Fig. 5b) at both 24 h and 7 days post-reperfusion was observed in ATTM-treated animals. SOD activity (Fig. 5c) was also superior with ATTM-treatment at 7 days (*p* < 0.05) while catalase (Fig. 5d) showed significantly (*p* < 0.01) greater activity at 24 h post-reperfusion. No differences in brain tissue levels were seen for the early-onset cytokine, TNF-α (Fig. 6a). However, ATTM-treatment blunted the late (7-day) pro-inflammatory response, with significant reductions in brain IL-1β (*p* < 0.05), IL-6 (*p* < 0.01), and NOx (*p* < 0.01) levels (Fig. 6b–d, respectively).

## Discussion

This study demonstrates significant neuroprotective effects of ATTM given at reperfusion following tMCAO, the most commonly utilized preclinical ischemic stroke model. In addition to improving histological outcomes, we demonstrated a significant improvement in behavioral functionality that was associated with improved antioxidant enzyme capacity, reduced oxidative damage, and a blunted late pro-inflammatory cytokine response in brain tissue. While we used a pharmacological approach to modify metabolism, therapeutic hypothermia is a frequently studied alternative that reduces oxidative metabolism and limits ROS production in preclinical injury models [38]. However, this approach has shown inconsistent outcome benefits in clinical studies of ischemia/reperfusion injury [39]. This may relate to the duration of time—often hours—needed to reach target temperatures. By contrast, our results here suggest ATTM could offer a rapid, straightforward, and potentially more effective alternative.

Basic sulfur salts, the so-called sulfide generators, showed initial promise as putative therapies [15], with efficacy demonstrated in several preclinical ischemia/reperfusion rodent models [17–19]. However, upon dissolution, these basic salts release all of their sulfur as sulfide in a near-instantaneous fashion with resulting implications for safety and efficacy [40]. Additionally, on translation to larger mammalian species, including sheep, pigs, and humans, the impact of the basic salts on metabolism diminishes [41]. The subsequent development of slow-release sulfide donors and sulfide-hybrid drugs aimed to deliver sulfide in a more controlled manner, allowing steady-state release over longer periods.

We previously described the unique sulfide-releasing properties of ATTM [23], a treatment used over many decades as an oral copper chelator to treat Wilson’s disease and, more recently, investigated as an anti-cancer agent [42]. We could demonstrate organ protection and outcome benefit in reperfusion models of myocardial infarction, global brain injury, and resuscitated hemorrhage [23], and were thus keen to extend these results to a relevant preclinical stroke model. Importantly, we have recently provided evidence that ATTM acts as a targeted therapy [23]. Its sulfide release profile is dependent on several biologically relevant factors; more acidic conditions and the presence of thiols (e.g., glutathione), both of which are encountered intracellularly, help ensure that sulfide is preferentially released within this compartment. Furthermore, we recently demonstrated that ATTM utilizes non-selective plasma membrane ion (anion-exchanger [AE]-1) channels to gain access to intracellular compartments [25]. Therefore, the favorable stability profile of ATTM that allows uptake of intact drug via the AE-1 protein into cells, followed by its controlled release of sulfide in intracellular compartments, provides attractive features that distinguish this sulfide donor from other compound classes.

During ischemia, the tissue area at risk will contain both salvageable and non-salvageable cells. While the new stroke reperfusion era has revolutionized management by improving short- and long-term outcomes, the necessary revascularization-induced reperfusion injury has become a major therapeutic target [43]. During initial reperfusion, salvageable cells are subjected to a nutrient supply in excess of their metabolic requirements, and this disproportionate substrate provision drives mitochondrial respiration to a level that generates large quantities of damaging ROS [26]. By modulating this response with a short-term inhibitor of mitochondrial cytochrome C oxidase, it is possible to achieve a level of metabolism that supports cell viability and functionality, yet without the detrimental over-production of ROS. We previously demonstrated that ATTM is able to downregulate oxidative metabolism (ex vivo and in vivo) in a dose-dependent manner [23]. Furthermore, an in vitro anoxia/reoxygenation study showed that ATTM could improve cell viability and reduce (mitochondria-specific; MitoSox^TM^) superoxide levels [23]. As reperfusion injury evolves beyond the initial ROS burst, several downstream factors come into play. Overwhelmed innate antioxidant defenses can lead to sustained, excessive levels of ROS, with subsequent oxidative damage and initiation of pro-inflammatory cascades that generate further ROS production [12]. We found clear evidence here that ATTM treatment, commenced at the time of reperfusion, was able to modify these responses. An improved anti-oxidant enzyme capacity and decreased oxidative damage to both proteins and lipids support our claims.

An important inflammatory pathway involved in ischemia/reperfusion injury is NLRP3 activation by mitochondria-derived ROS, with production of IL-1β [44]. Sulfide decreases NF-κB translocation [45], preventing NLRP3 activation and cytokine production. We too documented a reduction in pro-inflammatory cytokines that occurred downstream of our observed effects on redox balance in brain tissue. Notably, direct anti-inflammatory effects of ATTM have also been reported [46, 47]. These studies necessarily assumed that copper chelation was the primary mechanism for this action. However, given that it takes several weeks of repeated dosing to reduce systemic copper levels [48], (and that we used only one intravenous dose of ATTM acutely) a sulfide-mediated reduction in mitochondria-derived oxidative stress (that subsequently limits oxidative damage and inflammatory responses), is a more plausible mechanism. It is further notable that sulfide can increase the activities of Mn-SOD and Cu/Zn-SOD, both by upregulating their gene expression and by direct interaction with Cu/Zn-SOD [49]. Conversely, ATTM has been identified as a SOD inhibitor, a mechanism believed partially responsible for its efficacy as an anti-cancer agent [50]. It hence appears contradictory that ATTM-treatment induced an increase in SOD (and catalase) activity after tMCAO. However, the impact of ATTM as a SOD inhibitor requires depletion of copper that, as noted above, is unlikely to be relevant in our study.

## Notes and limitations

Although our work here is solely preclinical, we aimed to design our experiments with translation to the clinical setting in mind. We administered ATTM just prior to reperfusion, as would be feasible in patients; in doing so, we have avoided a preconditioning-like effect often prominent with drug pre-treatment in laboratory studies and limited any potential pharmacological impact of our intervention on the ischemia component of our insult.

Assessing functional and long-term outcome measures are key components in preclinical rodent models [13]. This is particularly relevant to tMCAO since, by contrast to permanent MCAO, transient occlusion and reperfusion induces a more delayed phenotype [51]. Accordingly, and in addition to the commonly used (acute) infarct size outcome [13], we included delayed measurements of legion area and behavioral testing that covered two distinct functionality tests. We chose the tMCAO model for this work as this is the most commonly used in preclinical stroke research. We acknowledge that we have not used aged or comorbid animals that more closely mirror the phenotype of stroke patients [52]. However, in an era where efficacy demonstrated in rodent models can draw a degree of apathy borne from past failures (particularly in stroke research), we consider that some important differences with other (initially) promising preclinical therapies should be noted. ATTM has been administered extensively to large mammals and humans over many decades, and thus is considered to have an excellent safety profile. Accompanied by the (clinically relevant) timing of our intervention, our long-term follow-up, demonstration of histological and functional improvement, and efficacy shown across numerous reperfusion-based scenarios in two distinct laboratories (Brazil and UK), we consider that these factors provide much encouragement of potential success where other approaches have fallen short.

The new stroke reperfusion era dictates that outcomes following revascularization are defined by adequate cerebral blood flow. As such, the STAIR (Stroke Therapy Academic Industry Round-table) criteria stipulate that cerebral blood flow (CBF) should be measured in all preclinical studies [52]. Unfortunately, we did not have the technology available to perform these measurements but we remain confident in our results on two counts: (i) we have previously [23] seen no impact on cardiac output, contractility, or global hemodynamics at the ATTM-dose level used here and (ii) all animals showed some evidence of focal damage which is consistent with a fall in CBF, and we would expect the median quantity of damage from the ischemia component of our insult to be similar in both groups.

A key factor underpinning the putative success of ATTM in neurological conditions is the ability of the molecule to penetrate the blood-brain barrier (BBB). Although we are currently unable to measure brain ATTM levels, we presumed the molecule can cross the BBB on three counts. First, putative BBB transport enabled efficacy in our previous global brain injury model [23] and in our findings reported here. Second, following administration of ATTM to sheep, molybdenum, the transition metal core of ATTM, is selectively distributed and retained by many organs, including the brain [53]. Third, we have noted a role for the AE-1 channel in the intracellular uptake of ATTM in human erythrocytes [25]. The AE-1 protein is also expressed at both luminal and abluminal membranes of endothelial cells of the BBB [54]. The participation of this ion channel in facilitating ATTM access to the brain and other target tissues will be further investigated.

Finally, we concede that we used fairly coarse measures of antioxidant enzyme activities here, and further work should address the impact of each dismutase isoform and activated catalase compounds. Other possible antioxidant mechanisms could also be explored that include a potential decreased expression of gp91phox [55], a direct ROS scavenging effect by sulfide [55], and intracellular redox recycling following interaction of ATTM with oxidized glutathione [25].

## Conclusion

ATTM, given at reperfusion, enabled significant neuroprotection at 24 h and 7-days post-insult with improvements in histological, functional, and molecular outcome measurements. These findings, allied with our extensive preclinical characterization [23, 25] and the known safety profile of this compound having been used for many decades in humans, provide much encouragement for the repurposing and development of ATTM as a novel adjunct to revascularization for the treatment of stroke.

## Supplementary information

**Additional file 1:** Supplementary Figure 1: Brain slices. Representative selection of original brain slices used for the quantification of infarct size.

## Abbreviations

AE-1: Anion exchanger 1
ARRIVE: Animal Research: Reporting of In vivo Experiments
ATTM: Ammonium tetrathiomolybdate
BBB: Blood-brain barrier
CAT: Catalase (units of activity)
CCO: Common carotid artery
DAMP: Damage-associated molecular pattern
ECA: External carotid artery
ELISA: Enzyme-linked immunosorbent assay
ICA: Internal carotid artery
IL: Interleukin
MCA: Middle cerebral artery
MCAO: Middle cerebral artery occlusion
MDA: Malondialdehyde
NED: Naphthylethylenediamine dihydrochloride
NF-κB: Nuclear factor-κB
NOx: Nitric oxide products
OA: Occipital artery
OD: Optical density
PVC: Polyvinyl chloride
ROS: Reactive oxygen species
SOD: Superoxide dismutase
STAIR: Stroke Therapy Academic Industry Round-table
TBARS: Thiobarbituric acid-reactive substances
TNF-α: Tumor necrosis factor-α
tMCAO: Transient middle cerebral artery occlusion
TTC: 2,3,5-triphenyltetrazolium chloride
