# Supplementary figures and images for: Neuroprotective effects of ammonium tetrathiomolybdate, a slow-release sulfide donor, in a rodent model of regional stroke
Source: Intensive Care Med Exp. 2020 Apr 9;8:13. doi: 10.1186/s40635-020-00300-8 (PMC7145883; doi:10.1186/s40635-020-00300-8)

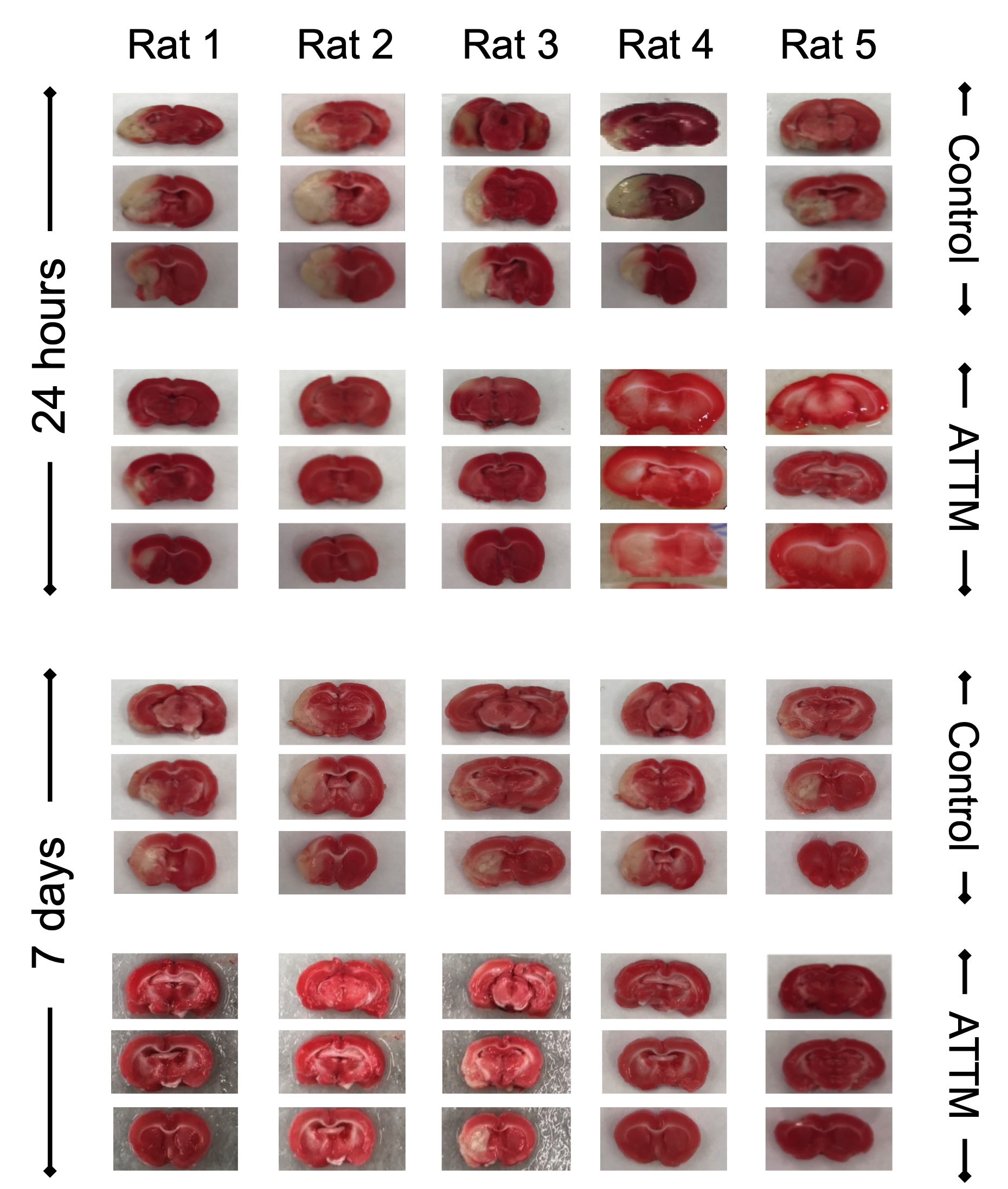

Supplement: Supplementary file 1 — Additional file 1: Supplementary Figure 1: Brain slices. Representative selection of original brain slices used for the quantification of infarct size. [file 40635_2020_300_MOESM1_ESM.tiff]
